# Sex-Specific Differences in Resolution of Airway Inflammation in *Fat-1* Transgenic Mice Following Repetitive Agricultural Dust Exposure

**DOI:** 10.3389/fphar.2021.785193

**Published:** 2022-01-13

**Authors:** Arzu Ulu, Jalene V. Velazquez, Abigail Burr, Stefanie N. Sveiven, Jun Yang, Carissa Bravo, Bruce D. Hammock, Tara M. Nordgren

**Affiliations:** ^1^ Division of Biomedical Sciences, School of Medicine, University of California, Riverside, Riverside, CA, United States; ^2^ Department of Entomology and Nematology, University of California Davis Comprehensive Cancer Center, University of California, Davis, Davis, CA, United States; ^3^ Department of Environmental and Radiological Health Sciences, Colorado State University, Fort Collins, CO, United States

**Keywords:** *fat-1* transgenic mice, omega-3 fatty acids, agricultural dust, resolution of inflammation, sex

## Abstract

In agriculture industries, workers are at increased risk for developing pulmonary diseases due to inhalation of agricultural dusts, particularly when working in enclosed confinement facilities. Agricultural dusts inhalation leads to unresolved airway inflammation that precedes the development and progression of lung disease. We have previously shown beneficial effects of the omega-3 polyunsaturated fatty acid (ω-3 PUFA) DHA in protecting against the negative inflammatory effects of repetitive dust exposure in the lung. Dietary manipulation of pulmonary disease risk is an attractive and timely approach given the contribution of an increased ω-6 to ω-3 PUFA ratio to low grade inflammation and chronic disease in the Western diet. To prevent any confounding factors that comes with dietary supplementation of ω-3 PUFA (different sources, purity, dose, and duration), we employed a *Fat-1* transgenic mouse model that convert ω-6 PUFA to ω-3 PUFA, leading to a tissue ω-6 to ω-3 PUFA ratio of approximately 1:1. Building on our initial findings, we hypothesized that attaining elevated tissue levels of ω-3 PUFA would attenuate agricultural dust-induced lung inflammation and its resolution. To test this hypothesis, we compared wild-type (WT) and *Fat-1* transgenic mice in their response to aqueous extracts of agricultural dust (DE). We also used a soluble epoxide hydrolase inhibitor (sEH) to potentiate the effects of ω-3 PUFA, since sEH inhibitors have been shown to stabilize the anti-inflammatory P450 metabolites derived from both ω-3 and ω-6 PUFA and promote generation of specialized pro-resolving lipid mediators from ω-3 PUFA. Over a three-week period, mice were exposed to a total of 15 intranasal instillations of DE obtained from swine confinement buildings in the Midwest. We observed genotype and sex-specific differences between the WT vs. *Fat-1* transgenic mice in response to repetitive dust exposure, where three-way ANOVA revealed significant main effects of treatment, genotype, and sex. Also, *Fat-1* transgenic mice displayed reduced lymphoid aggregates in the lung following DE exposure as compared to WT animals exposed to DE, suggesting improved resilience to the DE-induced inflammatory effects. Overall, our data implicate a protective role of ω-3 FA in the lung following repetitive dust exposure.

## 1 Introduction

Agricultural workers are at increased risk for developing various respiratory diseases including chronic bronchitis, asthma, and COPD, due in part to exposure to respirable organic dusts associated with these environments (Von Essen and Romberger, 2003; Nordgren and Charavaryamath, 2018; Sigsgaard et al., 2020). Individuals that work in concentrated animal feeding operations, such as those housing swine, have appreciably increased risk for negative lung health outcomes (Iversen and Dahl, 2000; Kirkhorn and Garry, 2000; Pedersen et al., 2000; May et al., 2012; Pavilonis et al., 2013; Guillien et al., 2016; Nordgren and Bailey, 2016; Nordgren and Charavaryamath, 2018). Therapeutic options for affected individuals are limited, with no current treatments to reverse lung function decline associated with these ailments (American Thoracic, 1998; Kirkhorn and Garry, 2000; Kachan et al., 2012; Hoppin et al., 2014). Thus, novel treatment strategies that harness and/or promote reparative processes in the lung are necessary.

It is increasingly appreciated that inflammation resolution is an active process and regulated by a variety of pathways and mediators, some of which involve omega-3 (ω-3) and omega-6 (ω-6) polyunsaturated fatty acids (PUFA) (Levy and Serhan, 2014; Serhan et al., 2014; Hammock et al., 2020). As ω-3 PUFA are essential fatty acids that cannot be synthesized *de novo* by humans, dietary consumption of ω-3 PUFA dictates the tissue availability for these fatty acids and mediators derived from them. In a typical Western diet, ω-3 PUFA intakes are below recommended guidelines, while ω-6 PUFA intakes are high (Thompson et al., 2019). Conversely to ω-3 PUFA, ω-6 PUFA are metabolized into lipid mediators (e.g., leukotrienes, thromboxane, prostaglandins) that are largely involved in the induction of inflammatory processes (Calder et al., 2020). Thus, individuals consuming a diet with a high ω-6: ω-3 PUFA ratio may be at increased risk for inadequate control of inflammatory processes, with increased substrate to produce pro-inflammatory lipid mediators and a dearth of substrate for the production of specialized pro-resolving mediators (SPM).

We have recently assessed the efficacy of dietary supplementation with the ω-3 PUFA docosahexaenoic acid (DHA) on altering the lung inflammatory response and recovery following acute and repetitive organic dust exposure (Nordgren et al., 2014; Dominguez et al., 2020; Ulu et al., 2021a). Mice were fed a mouse chow supplemented with DHA for four consecutive weeks prior to challenge with a single DE exposure (acute model) or DE challenge over 3 weeks (repetitive model). In these investigations, we identified impacts of a high DHA diet on lung inflammation, including alterations in macrophage activation, that were overall protective against the deleterious impacts of DE exposure. However, these studies were limited in that they only assessed the impacts of one ω-3 PUFA, DHA, on male sex and on a limited dietary regimen of 4–7 weeks (Dominguez et al., 2020; Ulu et al., 2021a). Sex-specific differences in respiratory symptoms are observed among the asthmatic individuals and agricultural workers with asthma being more common in women than men and respiratory symptoms being more prevalent in men than women among the farmers (CDC, 2019; Fix et al., 2020). To better assess the impacts of a high ω-3 PUFA diet on the lung inflammatory response to DE and achieve a total tissue ω-6: ω-3 PUFA ratio of ∼1:1 that is considered ideal, we have now utilized the *Fat-1* mouse transgenic model (Kang et al., 2004; Bilal et al., 2011) to better assess the sex-specific impacts of ω-3 PUFA on DE-induced inflammation. These mice express the *Caenorhabditis elegans (C. elegans)* fatty acid desaturase gene that converts ω-6 PUFA to ω-3 PUFA, thus yielding an overall tissue ratio of ∼1:1. We hypothesized that use of this model would enhance the protective effects identified in initial studies utilizing only DHA supplementation, while also overcoming study limitations that plague fatty acid supplementation investigations, including ambiguous outcomes due to different fatty acid sources, purity, doses, and duration of supplementation (Bradberry and Hilleman, 2013). In addition, we have also tested a strategy to further enhance the efficacy of ω-3 PUFAs through the use of a therapeutic inhibitor of soluble epoxide hydrolase (sEH) an enzyme that metabolizes lipid mediators such as SPM into inactive or less active forms (Ulu et al., 2013; Ulu et al., 2014; Ulu et al., 2016; Dileepan et al., 2019).

Through these investigations, we have clarified a role for ω-3 PUFA in regulating the initiation of lung inflammation following DE inhalation and identified differentially regulated genes in repair and recovery following these exposures. These studies warrant consideration of ω-3 PUFA supplementation as a complementary therapeutic strategy for protecting against the deleterious lung diseases associated with environmental dust exposures, such as those experienced by agriculture workers.

## 2 Materials and Methods

### 2.1 Preparation of Aqueous Dust Extracts

Settled dusts in closed swine confinement facilities (Nebraska) were collected one foot above the ground and kept at −20°C. Dust extracts were prepared as previously described (Romberger et al., 19852002). Briefly, 5 g dust was mixed with 50 ml Hank’s Balanced Salt Solution at room temperature for 1 h. The mixture was then centrifuged at 2,500 rpm for 20 min at 4°C, supernatant was centrifuged one more time and resultant supernatant was sterile filtered using a 0.22 μm filter. Extracts were aliquoted, labeled as 100% dust extract (DE) and kept frozen at −20°C. A 12.5% DE solution was prepared for use in mouse intranasal instillations by diluting the 100% extract with sterile saline. Detailed analyses of the DE have been performed previously (Poole et al., 2010; Boissy et al., 2014; Romberger et al., 2015). A previous study compared immune response to the agricultural dust administered *via* intranasal instillation and 100 µg LPS challenge in mice (Poole et al., 2011), which has been estimated to be approximately 250× more than the LPS in 12.5% DE. In this same study, the mean endotoxin levels have been reported to be 0.384 μg/ml. Given this finding, when we administer 50 µL DE *via* intranasal route, this would correspond to approximately 20 ng LPS. In addition, other studies report respirable LPS levels to be between 14–129 EU/mL (one EU is approximately 0.1–0.2 ng/ml) (Demanche et al., 2009).

### 2.2 Animals and Dust Exposure Studies

All animal protocols were approved by the Institutional Animal Care and Use Committee of the University of California, Riverside. Male and female 10–12-week-old C57BL/6 WT (WT) and *Fat-1* transgenic mice [C57BL/6-Tg (CAG-*Fat-1*)1Jxk/J] were purchased from Jackson Laboratories and used to obtain a mouse colony. Breeding pairs were set as *WT x Fat-1* or *Fat-1 x Fat-1* to obtain both the WT and *Fat-1* genotypes. Mice had *ad libitum* access to food and water. Male and female mice were housed in separate cages with five mice/cage. Male and female pups reaching the age of 6–8 weeks were administered the sEH inhibitor TPPU [1-trifluoromethoxyphenyl-3-(1-propionylpiperidin-4-yl) urea] in their drinking water at 1 mg/kg dose for 1 week before intranasal dust exposure commences. For the repetitive dust exposure model, mice were instilled *via* the intranasal route with 50 µL of 12.5% DE for three-weeks (a total of 15 instillations, 5 days/week) as published before (Poole et al., 2009; Nordgren et al., 2015; Nordgren et al., 2019). Instillations were performed under light anesthesia using isoflurane.

### 2.3 Assessment of Airway Inflammation

#### 2.3.1 Enumeration of Infiltrating Immune Cells Into the Lung

At the end of the three-week period, mice were euthanized, and trachea were cannulated to obtain bronchoalveolar lavage fluid (BALF) from each mouse. Collection of BALF included three times washing with 1 ml PBS each time. All washes were centrifuged at 1,200 rpm for 5 min. While the first wash was kept separate, the second and third washes were combined before centrifugation. The supernatant from the first wash was aliquoted and stored in −80°C for cytokine profiling. The pelleted cells obtained from all the washes were combined and counted. Cytospin slides were prepared using 100,000 cells, stained with Diff-Quik kit (Siemens, Newark, DE) and differential cell counts were obtained as described before (Nordgren et al., 2015).

#### 2.3.2 Lung Histopathology

For histopathological assessments, lungs were inflated with 10% buffered formalin at 15 cm pressure. The same mouse lungs that were lavaged with PBS to obtain BALF was used for histology. Fixed lungs were transferred into 70% ethanol and then shipped to UC Irvine Pathology Research Services Core for paraffin embedding, sectioning, and Hematoxylin and Eosin (H and E) staining. The observer was blinded to the identity of each slide. A lymphoid aggregate was defined as close aggregation of ≥20 lymphocytes. Alveolar cellularity was evaluated by the number of cells in the alveolar spaces in the lung parenchyma in a total of five images obtained throughout the whole lung using 40× objective with 150% optical zoom. The resulting five values were averaged per tissue section. Each histopathological evaluation was represented as percentiles and a score between 0-to-4 was assigned for each percentile.

### 2.4 Lung NanoString Gene Expression Analysis

A mouse NanoString Immunology panel (NanoString Technologies, Seattle, WA, United States) was purchased for direct counting of 561 RNA transcripts using a nCounter Sprint Profiler. Each mouse lung was immediately put into 1 ml of RNA Later, kept at 4°C overnight, then stored in RNA Later at −80°C until RNA extraction. A total of three male mouse lungs per group obtained from three independent studies were thawed for RNA extraction. After lung samples were rinsed in sterile PBS, they were homogenized in 1 ml of Trizol using a 7 cm polypropylene pellet pestle in a microtube, then the extraction was performed as per manufacturer’s instructions using a PureLink RNA mini kit (Invitrogen, Carlsbad, California, United States). RNA integrity number was obtained for each sample (ranged from 7–9 on a scale of 1–10) at the UC Riverside Institute for Integrative Genome Biology Core Facility using an Agilent Bioanalyzer 2,100 (Agilent Technologies, Santa Clara, California, United States). Samples were prepared by a 16-h hybridization step of 50 ng RNA with the codeset probe provided in the Immunology panel. At the end of the hybridization, samples were diluted with nuclease-free water to 35 μL, and 32 µL of each sample was loaded onto a nCounter Sprint Cartridge. Given each cartridge can hold up to 12 samples, a total of 24 samples were run on two cartridges. All samples passed the QC test without any QC flags. The data resulting from each run were combined and analyzed together using nSolver 4.0 and NanoString Advanced analysis. On the nSolver software, gene expression data were normalized using ten housekeeping genes that showed strong correlation with each other, these included *Rpl19, Alas1, Ppia, Oaz1, Sdha, Eef1g, Gusb, Gapdh, Hprt and Tbp.* Heatmaps were generated using the agglomerative clustering analysis in nSolver software (nSolver 4.0, NanoString Technologies, Seattle, WA, United States, User Manual). For the advanced analysis, at least three housekeeping genes whose expression correlated well with each other (*Alas1, Ppia, Tbp and Tubb5*), and thus ideal for normalization of the data were used to normalize the raw data (NanoString, MAN-C0011-04 Gene Expression Data Analysis Guidelines), and a 77 transcript counts were taken as “count threshold”, which is two times the highest background-to-noise ratio (average of negative controls/sample + 2*standard deviation of negative controls/sample). Advanced analysis produced differential expression analysis, gene set analysis, and pathway scores. Differential expression outcomes identified the top 20 most upregulated genes among all the treatments, while gene set analysis displayed which pathways those most upregulated genes are related to.

To further explore the protein-protein interactions among differentially regulated proteins, we used the STRING database (https://string-db.org) of genes that were statistically significant based on the unadjusted p-values (Szklarczyk et al., 2021). Genes with low counts (counts < 79) were not included in any of the analyses. Raw and normalized NanoString data are deposited to https://www.ncbi.nlm.nih.gov/geo/info/spreadsheet.html.

### 2.5 Statistical Analyses

GraphPad Prism software (Prism 9) was utilized to perform two-way ANOVA tests and version eight was used to perform three-way ANOVA on data to determine the main effects of exposure and treatments, and post-hoc comparisons were performed to reveal significant differences among all the groups. Differences between groups were considered significant if the *p* value ≤ 0.05. Data are represented with mean ± standard error of the mean on all figures.

## 3 Results

### 3.1 Effects of Elevated Tissue Levels of ω-3 Fatty Acids on Dust-Induced Airway Inflammation

#### 3.1.1 Infiltrating Immune Cells and Pro-Inflammatory Cytokines

**FIGURE 1:**
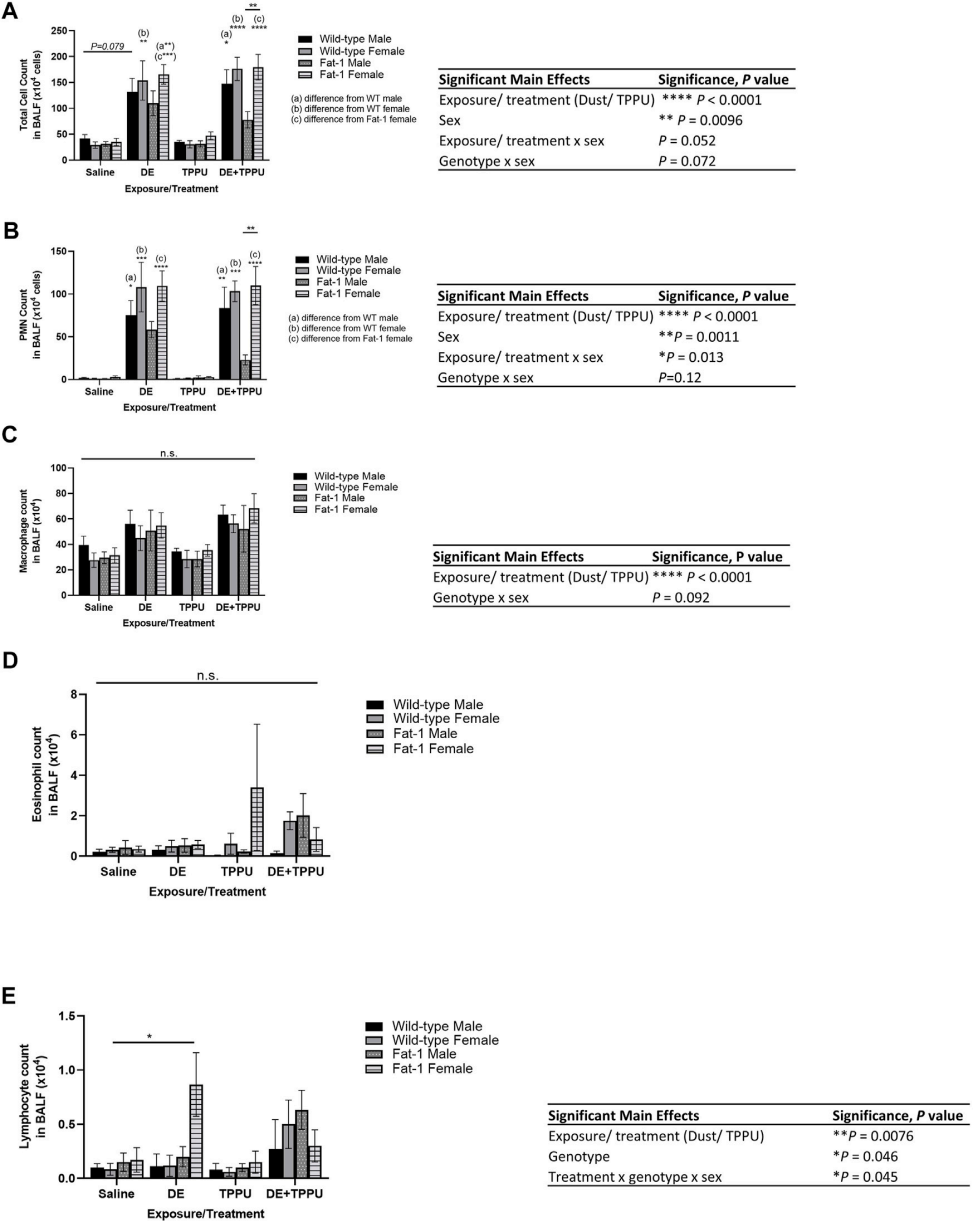
Genotype and sex-specific changes in infiltrating immune cells in the bronchoalveolar lavage fluid after a three-weeks repetitive dust exposure with or without TPPU treatment. **(A)** Total infiltrating cell counts, **(B)** neutrophil count, **(C)** macrophage count, **(D)** eosinophil count, **(E)** lymphocyte count; *n* = 6–8 mice WT male (except for WT TPPU n = 5), *n* = 6–8 WT female, *n* = 6 *Fat-1* male, *n* = 6–7 *Fat-1* female. Significant main effects of three-way ANOVA are shown. Statistical significance is shown with letters: **(a)** significantly different from WT male, **(b)** significantly different from WT female, **(c)** significantly different from *Fat-1* male, **(d)** significantly different from *Fat-1* female. Data are mean ± SEM. *p < 0.05, **p < 0.01, ***p < 0.001, ****p < 0.0001.

To test the hypothesis that elevated tissue levels of ω-3 fatty acids are protective against agricultural dust induced lung inflammation and improve inflammation resolution, we compared WT and *Fat-1* transgenic mice for their response to three-weeks repetitive dust exposure. As expected, mice exposed to aqueous dust extracts for the duration of three-weeks (5 instillations/week) had 2-3-fold increase in total BALF cell counts as compared to their corresponding saline controls. First, we examined the main effects of exposure, genotype, sex and TPPU treatment on the total infiltrating immune cell counts (Figure 1A). We found statistically significant differences among the groups that are driven by DE exposure, TPPU treatment (F = 51.2, *p* < 0.0001) and sex (F = 3.028, *p* = 0.0096). Further statistical analysis of the total cell counts also revealed that both sex and genotype plays a role in response to DE exposure and TPPU treatment (interaction between exposure/treatment x sex, F = 3.463, *p* = 0.052; genotype x sex, F = 1.417, *p* = 0.073). We observed similarly significant results for the infiltrating neutrophils but not for macrophages (Figures 1B,C). This increase in neutrophil numbers is consistent with the cellular signature of repetitive DE exposure (Nordgren et al., 2014; Nordgren et al., 2013; Ulu et al., 2021b; Warren et al., 2017). With regards to eosinophils, we did not detect any differences among the groups (Figure 1D). Lymphocyte counts showed a statistically significant increase only in *Fat-1* females that were exposed to dust. These counts were lower in the presence of TPPU; however, this difference did not reach significance (Figure 1E). All the significant main effects and relevant multiple comparisons are shown in each figure. When administered alone, TPPU did not affect any immune cell infiltration into the lung, which is consistent with previously reported homeostatic effects of sEH inhibition (Morisseau and Hammock, 2013; Vanella et al., 2015; Kuo et al., 2019). At the dose we administered (1 mg/kg), TPPU reaches plasma concentrations of >50-fold of the mouse IC_50_ of 2.8 nM, suggesting good target engagement (Supplementary Figure S1). Among the DE-exposed mice, TPPU was most effective in reducing the number of infiltrating cells in *Fat-1* male mice (Figures 1A,B). While this decrease with TPPU was not significant for total infiltrating neutrophils (*p* = 0.9, *Fat-1*+DE male mice vs. *Fat-1* DE + TPPU), we observed a striking difference between the *Fat-1* male versus *Fat-1* female mice receiving the TPPU treatment during dust-exposure (*p* = 0.032 for total infiltrating cells and *p* = 0.0029 for neutrophils between *Fat-1* male + DE + TPPU vs. *Fat-1* female + DE + TPPU). Since TPPU can stabilize the P450 metabolites derived from the ω-3 fatty acids, EPA (eicosapentaenoic acid) and DHA and also promote generation of SPMs, these data suggest that elevated ω-3 fatty acids could be stabilized with a sEH inhibitor to contribute to the decrease in pro-inflammatory cell influx into the lung in male sex.

**FIGURE 2:**
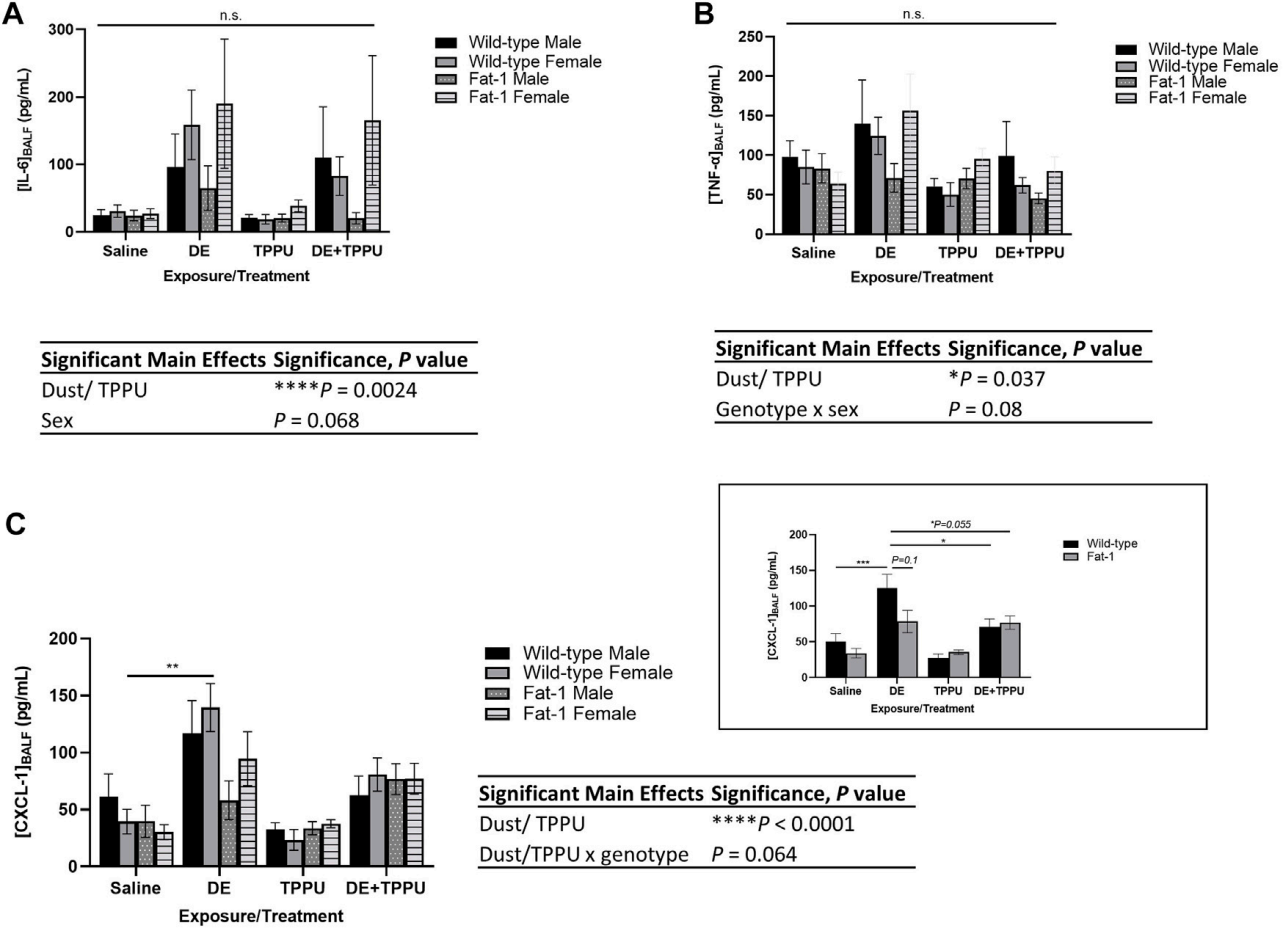
Effects of three-weeks repetitive dust exposure on pro-inflammatory cytokine levels in the bronchoalveolar lavage fluid with or without TPPU treatment. **(A)** IL-6, *n* = 5–7 mice WT male, *n* = 6–8 WT female, *n* = 5–7 *Fat-1* male, *n* = 6–7 *Fat-1* female; **(B)** CXCL-1, *n* = 5–8 mice WT male, *n* = 5–6 WT female, *n* = 4–6 *Fat-1* male, *n* = 5–7 *Fat-1* female; **(C)** TNF-α, *n* = 5–8 mice WT male, *n* = 5–7 WT female, n = 5-6 *Fat-1* male, *n* = 5–7 *Fat-1* female. The inset shows data from both female and male sexes, *n* = 11–13 for WT and *n* = 9–12 for *Fat-1* mice. Data are mean ± SEM. Significant main effects of three-way ANOVA are shown. Statistical significance is shown with letters: **(a)** significantly different from WT male, **(b)** significantly different from WT female, **(c)** significantly different from *Fat-1* male, **(d)** significantly different from *Fat-1* female. **p < 0.01, ***p < 0.001.

Similarly, pro-inflammatory cytokines in BALF decreased in *Fat-1* transgenic mice receiving TPPU among the other *Fat-1* mice exposed to dust (Figure 2). The three-way ANOVA analysis showed significant main effects of dust exposure/TPPU (F = 13.87, *p* = 0.0024) and a trend for the main effect of sex (F = 3.02, *p* = 0.068) on BALF IL-6 levels (Figure 2A). Regarding the BALF levels of TNF-α, we observed significant main effects of dust exposure or TPPU (F = 9.48, *p* = 0.037). For the neutrophil chemoattractant CXCL-1, we observed a statistically significant main effect of exposure/treatment (F = 29.8, *p* < 0.0001) and a statistical trend for interaction between dust exposure/TPPU x genotype (F = 5.4, *p* = 0.064). Since sex did not have a significant effect on CXCL-1 BALF levels, we also examined the data after combining both sexes. Similarly, we found a significant main effect of treatment (F = 30.36, *p* < 0.0001). When we investigated the specific differences among the groups, we found significant differences between WT + DE vs. WT + DE + TPPU as well as WT + DE vs. *Fat-1* + DE + TPPU groups (Figure 2C, inset). Similarly, dust-exposed *Fat-1* transgenic mice exhibited lower levels of CXCL-1 as compared to WT mice; however, this difference did not reach significance (*p* = 0.1).

#### 3.1.2 Effect of Dust Exposure on Lung Histopathology in *Fat-1* Transgenic Mice

**FIGURE 3:**
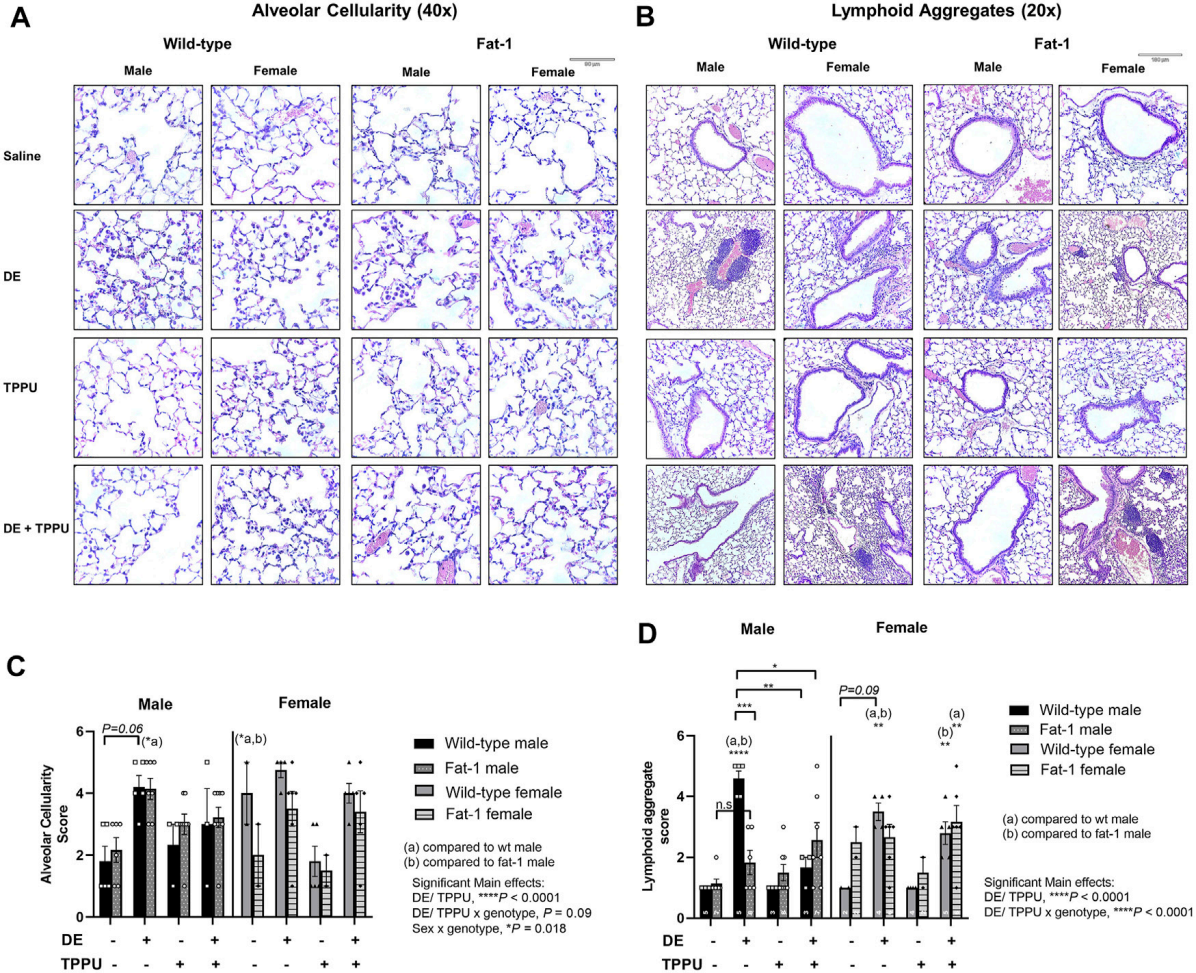
Representative images and scoring of lung histopathology in WT and *Fat-1* transgenic mice following 3 weeks exposure. **(A)** Alveolar cellularity, *n* = 3–5 mice WT male, *n* = 2–5 WT female, *n* = 5–9 *Fat-1* male, *n* = 2–6 *Fat-1* female; **(B)** lymphoid aggregate formation, *n* = 5–8 mice WT male, *n* = 5–6 WT female, *n* = 4–6 *Fat-1* male, *n* = 5–7 *Fat-1* female; **(C)** quantification and pathological scoring of the alveolar cellularity and **(D)** lymphoid aggregate formation. Sample sizes for each group were shown on each graph. **(a)** significantly different from WT male, **(b)** significantly different from WT female. Data are mean ± SEM. *p < 0.05, **p < 0.01, ***p < 0.001, ****p < 0.0001.

Lung histopathology was evaluated in H&E-stained paraffin-embedded tissue sections mounted on slides to evaluate dust-induced lung inflammation (Figure 3). We have previously shown that total number of lymphoid aggregates (defined as at least 20 closely aggregating cells) and alveolar inflammation evaluated as the number of macrophages in alveolar spaces increase with repetitive exposure to agricultural dust (Ulu et al., 2021b). The 2-3-fold increase in the number of BALF leukocytes following repetitive exposure to DE was reflected in approximately two fold increase in histopathological scores.

As expected, the number of macrophages in alveolar spaces increased approximately two-fold with three-weeks repetitive exposure to DE in both WT and *Fat-1* transgenic mice with a significant main effect of dust exposure and/or TPPU (F = 29.97, *p* < 0.0001) (Figures 3A,C). We also observed significant interaction between sex and genotype (F = 5.11, *p* = 0.018), which was reflected in a statistically significant difference in alveolar inflammation score in *Fat-1* males and WT females that were receiving the three-weeks DE exposure as compared to their saline control counterparts.

Similarly, we observed a significant main effect of DE and/or TPPU (F = 32.15, *p* < 0.0001) and their interaction with genotype (F = 16.95, *p* < 0.0001) on lymphoid aggregate formation (Figures 3B,D). In WT mice receiving the three-weeks DE exposure, the number of lymphoid aggregates significantly increased as compared to WT saline controls (*p* < 0.0001). Among the mice exposed to DE, *Fat-1* mice exhibited less lymphoid aggregates than the WT mice but only in the male sex (*p* = 0.0002). In addition, we observed a sex-dependent difference in TPPU treatment, which significantly reduced lymphoid aggregate formation in male mice receiving the three-weeks DE exposure regardless of the genotype (*p* = 0.0019, WT + DE male vs. WT + DE + TPPU male and *p* = 0.014, WT DE male vs. *Fat-1* + DE + TPPU male).

### 3.2 Changes in Expression of Genes Related to Immune Response in WT and *Fat-1* Transgenic Mice Following Repetitive Dust Exposure

**FIGURE 4:**
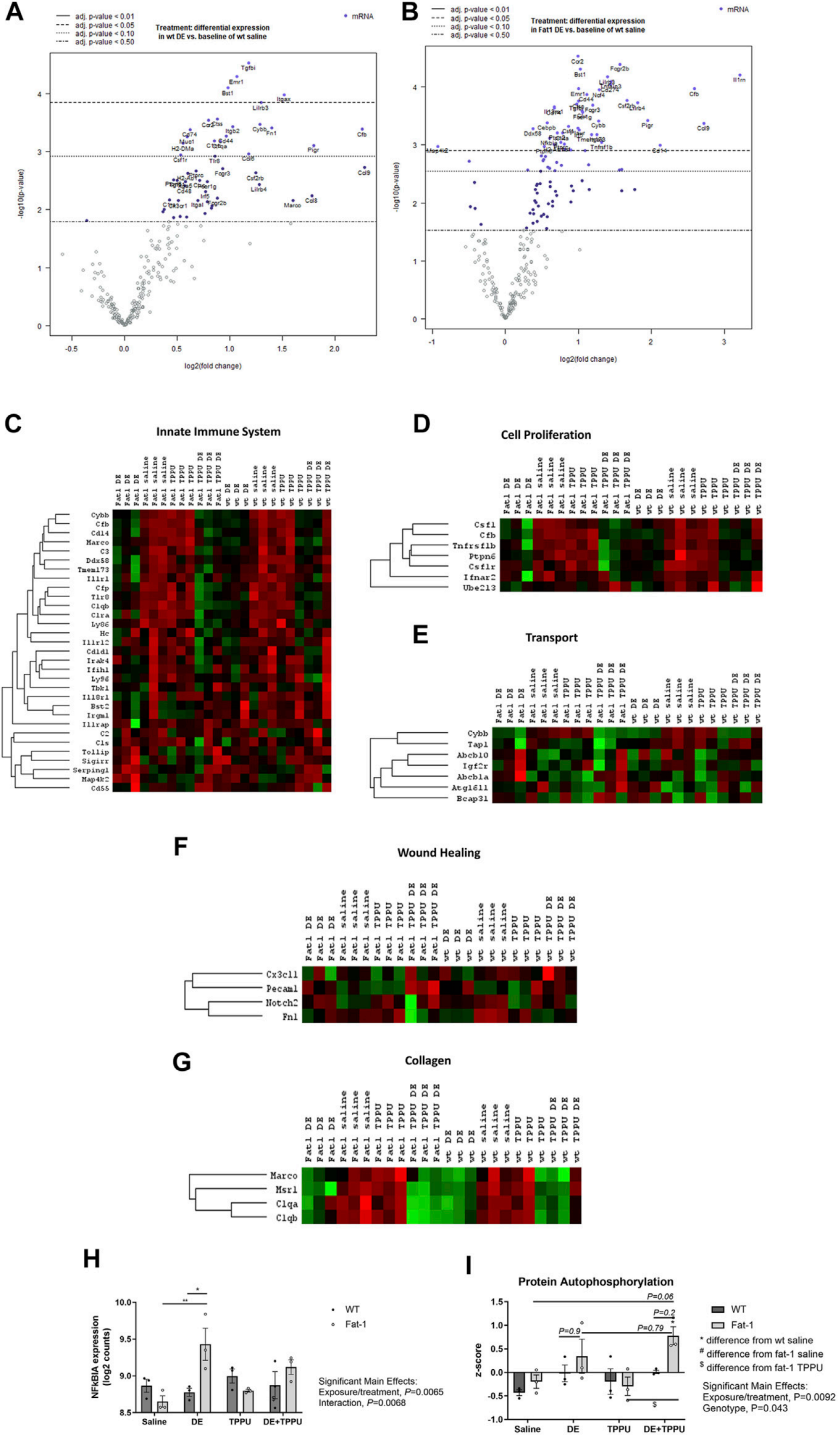
Changes in gene expression and related pathways altered after three-weeks repetitive dust exposure. Volcano plots showing differential expression of genes in **(A)** WT and **(B)** *Fat-1* transgenic mice as compared to WT saline controls. Heatmaps were generated for each sample for genes involved in **(C)** innate immune system, **(D)** cell proliferation, **(E)** transport, **(F)** wound healing and **(G)** collagen. **(H)** The top up-regulated gene, NFκBIA in *Fat-1* mice as compared to WT mice receiving the repetitive DE exposure. **(I)** Expression of genes coding for proteins involved in autophosphorylation. *n* = 3 male mice/group.

ω-3 fatty acids are known to regulate gene expression (Ulven and Holven, 2020). To elucidate differences in lung gene expression between the WT and *Fat-1* transgenic mice following repetitive dust exposure, we used a NanoString Mouse Immunology Panel as described in the Methods. We used only male sex for the gene expression study since we saw the most significant changes in the male sex as compared to the females. Following data acquisition, an automated QC test (quality control) was performed on nSolver software which showed that all samples passed this test with no warning flags. Then, samples were normalized to housekeeping genes and principal component analysis (PCA) of the data were performed. The most significantly altered genes after the three-weeks dust exposure are shown for both WT and *Fat-1* mice in volcano plots on Figures 4A,B as compared to WT saline controls. The overall significant changes among all the groups are summarized with the heatmap in Supplementary Figure S2. Among these genes, several gene sets included in the Immunology panel showed high PC1 scores and a clear clustering of the saline vs. DE-exposed groups regardless of genotype or treatment with TPPU, suggesting that DE exposure is the main driver of the observed changes (Figure 4). Some of the gene sets with high PC1 scores include innate immune system, cell proliferation, transport, wound healing, and collagen. (Figures 4C–G, and Supplementary Figure S3).

To assess the effects of genotype and TPPU treatment on gene expression, we performed additional analyses that allowed us to make direct comparisons between the groups. These included advanced analyses of the raw NanoString data by selecting WT + DE, *Fat-1* + DE, WT + DE + TPPU groups as reference instead of the WT saline group as selected in the initial analysis. When WT + DE group served as reference, nSolver software was able to directly compare WT + DE with *Fat-1* + DE group. The most significantly altered gene between the two genotypes was NFκBIA, which acts a NFκB inhibitor by binding and confining it to the cytoplasm (Figure 4H). We found a significant interaction between the exposure/treatment and genotype (F = 39.56, *p* = 0.0068) and close to statistical significance with DE exposure and TPPU treatment (F = 19.86, *p* = 0.065). The *Fat-1* transgenic mice had significantly higher log2 counts of NFκBIA as compared to *Fat-1* saline controls (*p* = 0.0061) and to that of WT mice receiving the repetitive DE exposure (*p* = 0.025). When looking at the significant unadjusted p values, among the top 20 differentially regulated genes, we found *Cd83, Tnfaip3, Il33, Xbp-1, Il4ra, S100a9, S100a8, Cebpb, Csf1, Il1rn, Ptpn2, Il10rb, Jak2, Il13ra1, Tmem173, Fcgr2b, and Ccrl2 to be upregulated in Fat-1 + DE, and Ahr and Cd2* to be down-regulated in *Fat-1* + DE as compared to WT + DE groups. With this analysis, we also examined the differentially regulated genes between the WT + DE *versus* WT + DE + TPPU groups, and we observed only the *LCP2 (Lymphocyte cytosolic protein 2)* gene to be upregulated based on the significant unadjusted p values. Next, we repeated the analysis selecting *Fat-1* + DE group as the reference which allowed us to compare gene expression in *Fat-1* + DE group to *Fat-1* + DE + TPPU. Based on the unadjusted p values, the top 20 upregulated genes in *Fat-1* + DE + TPPU included *Cxcr1, Cd2, Prkcd, H2-Dma, Itgb2, Fn1, H2-Eb1, Itgax, Csf1r, C1qb, Itga4, Lcp2, Ly86, Xbp-1, Irf8, Cd48, Npc1, Tlr8, C1qa, and Cd79b*. After identifying differentially regulated genes for the hypotheses we wanted to test, we entered these genes into the STRING Database to identify any possible interactions and biological pathways that these cluster of genes would be related to. Results from these analyses are shown on Table 1. As expected, DE exposure was consistent with pathways related to immune cell activation, cell proliferation, neutrophil aggregation, and antigen presentation regardless of mouse genotype. To our surprise, this analysis revealed a role for DE exposure in positively regulating hematopoietic stem cell migration as well as negative regulation of hippocampal neuron apoptotic processes. Interestingly, the main differences between the WT and *Fat-1* genotype were associated with response to macrophage colony stimulating factor, immune clearance, and neutrophil aggregation. Upon examining the different gene sets, we found that genes in the protein autophosphorylation pathway show a significant main effect of genotype (Figure 4I). The genes involved in this gene set include *Jak 1/2, Irak 1/2, Mapk14, Csf1r, Ptk2, Fyn, Prkcd, and Src.* With TPPU treatment, main differences appeared to be in positive regulation of T-cell differentiation in WT mice and positive regulation of PMN activation in *Fat-1* mice. Consistent with these changes in upregulated genes, downregulated genes also showed a similar pattern. A list of upregulated and downregulated genes is provided separately in the Supplementary Table S1.

**TABLE 1:** STRING Database analysis of the differentially regulated genes and their association with biological pathways.

	Upregulated genes	STRING database biological process	P	Downregulated genes	STRING database biological process	P
WT DE vs. WT saline	74	Pos reg hematopoietic stem cell migration	<1.0e-16	2	n/a	n/a
		Neg reg hippocampal neuron apoptotic process				
		Immune cell clearance				
Fat-1 DE vs. WT saline	99	Pos reg cellular response to macrophage colony stimulating factor	<1.0e-16	13	Protein localization to bicellular tight junction	2.11e-06
		Pos reg hematopoietic stem cell migration			Regulation of macrophage cytokine production	
		Pos reg growth factor dependent skeletal muscle proliferation			Regulation of ECM disassembly	
		Neutrophil aggregation				
		Antigen processing and presentation of exogenous peptide *via* MHC Class I				
Fat-1 DE vs. Fat-1 saline	80	Pos reg cellular response to macrophage colony stimulating factor	<1.0e-16	24	B-1 B cell homeostasis	7.86e-08
		Pos reg hematopoietic stem cell migration			IL-17 production	
		Neutrophil aggregation			Protein localization to bicellular tight junction	
Fat-1 DE vs. WT DE	25	Immune complex clearance	<1.0e-16	8	Neutrophil aggregation	n.s.
		Neutrophil aggregation			Immune complex clearance	
WT DE vs. WT DE + TPPU	1 (*LCP2*)	Positive regulation of T-cell differentiation	n/a	0	n/a	n/a
Fat-1 DE + TPPU vs. Fat-1 DE	31	Positive regulation of PMN activation	<1.0e-16	9	IL-33 mediated signaling pathway	0.000471
		Synapse pruning			Pos regulation of IgG, IL-5, IL-13 and IL-6 secretion	
Fat-1 DE + TPPU vs. WT DE + TPPU	43	Pos reg hematopoietic stem cell migration	<1.0e-16	4	Cell autonomous role of endothelial GTP cyclohydrolase1 and tetrahydrobiopterin in blood pressure regulation	0.109
		Neg reg hippocampal neuron apoptotic process				
		Antigen processing and presentation of exogenous peptide *via* MHC Class I				
		Macrophage colony-stimulating factor signaling pathway				

## 4 Discussion

The lungs are continually exposed to harmful stimuli found in the air, including environmental dusts, diesel exhaust particles, and smoke exposures. The ability of the airways to respond to these stimuli and repair damage caused by the exposures is vital to respiratory health, because unrepaired damage can lead to debilitating airway diseases (Ehrlich, 1980; Kirkhorn and Garry, 2000; Smit et al., 2014; Franklin et al., 2015; Nordgren and Bailey, 2016; Watkins et al., 2017). Long-term particulate matter exposures have been consistently linked to negative cardiovascular and lung health outcomes and increased mortality (Franklin et al., 2015; Watkins et al., 2017). Disease susceptibility caused by chronic inhalation of particulates is clearly evidenced by occupational exposures such as those seen in agriculture workers; exposures to livestock farming operations are consistently linked to increased respiratory symptoms and inflammatory lung disease in not only workers, but in individuals living in the surrounding communities, including children and adults (Radon et al., 2007; Pavilonis et al., 2013; Nordgren and Bailey, 2016; van Dijk et al., 2016; Baliatsas et al., 2017; Borlee et al., 2017; Rasmussen et al., 2017; Burkes et al., 2018). Approximately two-thirds of agriculture workers report respiratory disease; 50% of agriculture industry workers experience asthma-like symptoms (American Thoracic, 1998), 25–35% of individuals working in concentrated animal feeding operations experience chronic bronchitis (American Thoracic, 1998), and the prevalence of chronic obstructive pulmonary disease (COPD) among agriculture workers is doubled compared to nonfarming working control subjects (Guillien et al., 2016). Curative options are not available for these workers, with current therapeutic options aimed primarily at symptom management and the prevention of lung disease.

To improve treatment options for this population, studies investigating therapeutic mechanisms to stimulate endogenous lung tissue repair mechanisms are warranted. To this end, we have assessed the impacts of a low (∼1:1) ω-6: ω-3 PUFA total body tissue ratio on lung inflammation following repetitive exposure to inhaled environmental dusts, using a well-described mouse model of DE inhalation. In addition, we have explored the therapeutic utility of an sEH inhibitor, TPPU, in enhancing the impacts of high ω-3 PUFA tissue levels, including exploring its effects in regulating SPM levels during inflammation resolution. Taken together, our results indicate sex-dependent protective effects associated with elevated tissue levels of ω-3 fatty acids in reducing the number of infiltrating immune cells (i.e., neutrophils) into the lung, lower pro-inflammatory cytokine levels and reduced overall histopathology of the lung.

The results identified herein model a long-term dietary intake of high ω-3 PUFA and reduced intake of ω-6 PUFA that achieves an ideal ω-6:ω-3 PUFA ratio throughout the body as reviewed before (Lands et al., 2018). This was achieved through the use of the *Fat-1* transgenic mouse model; these mice express the *Fat-1* gene from *C. elegans* encoding an ω-3 FA desaturase, converting ω-6 PUFA to ω-3 PUFA, leading to tissue ω-6:ω-3 PUFA ratios of ∼1:1 (Kang et al., 2004). This model is advantageous because it overcomes several issues that limit diet- and supplementation-based experimental strategies; current clinical and preclinical studies have no standardization in terms of dose, duration, or source and quality of ω-3 PUFA, and each of these factors has implications on outcomes (e.g., due to differences in tissue incorporation, lipid peroxide or aldehyde formation) (Yang et al., 2019). These inconsistencies are considered leading factors for discrepancies in ω-3 PUFA study outcomes (von Schacky, 2014; Calder, 2010; Deckelbaum and Torrejon, 2012). The *Fat-1* mouse thus provides a preclinical model for assessing how elevated tissue levels of ω-3 PUFA and reduced ω-6 PUFA can influence health outcomes by minimizing variation based on supplementation, intake and absorption and distribution of ω-3 rich fatty acids. Using this model, we have found genotype and sex-specific differences in infiltrating cells (Figure 1), pro-inflammatory cytokines (Figure 2) and lung histopathology (Figure 3). The decreases in infiltrating PMNs and cytokines were consistent with each other within the *Fat-1* male sex. Among the swine farm workers, sex-specific differences in lung function and *TLR* gene polymorphisms, which is a toll like receptor activated by swine farm dust, have been reported before (Senthilselvan et al., 2009; Gao et al., 2018). Gao et al. found that lung function is worse in males with the *TLR9* gene polymorphism (rs187084) as compared to those males without the polymorphism, and females with the *TLR2* gene polymorphism (rs4696480) exhibit better functioning lungs as compared to those females without the polymorphism in swine farm full-time workers (Gao et al., 2018). Another study by Senthilselvan et al. found increased plasma levels of TNF-α in males without any *TLR4* gene polymorphisms (*TLR4 299/399* polymorphism), and in females with the *TLR4* gene polymorphism following 5 hours of swine farm exposure in naïve healthy subjects (Senthilselvan et al., 2009). We are not the first ones to report sex-specific differences in *Fat-1* mice; a recent study investigating the role of elevated tissue levels of ω-3 fatty acids in obesity-associated post-traumatic osteoarthritis also reported sex-specific differences in *Fat-1* transgenic mice (Kimmerling et al., 2020). It appears that such sex-dependent differences are disease model-specific where one sex in the *Fat-1* transgenic background exhibits a greater response than the other sex. Another study reported on sex-and age-specific differences of Resolvin D1 (RvD1, an SPM derived from DHA) levels in the retina. This study found sex-dependent differences in retinal levels of RvD1 in aged mice (24-months) as compared to young mice (three-months old), with aged male mice showing a larger decrease in RvD1 levels (Trotta et al., 2021). Sex-dependent changes in the expression of genes involved in fatty acid synthesis, steroids and drug metabolizing enzymes have been identified before (Waxman and Holloway, 2009). Some of those genes encode for enzymes involved in the metabolism of ω-3 and ω-6 PUFA, thus identifying sex-dependent differences can inform pharmacokinetics, bioavailability and treatment options related to ω-3 and ω-6 PUFA-derived lipid mediators. The sEH inhibitor TPPU was used in this study as an indicator that to the inflammation resolving epoxide metabolites of ω-3 lipids might be a partial explanation for their beneficial effect. It is attractive to consider sEH inhibitors or mimics of ω-3 fatty acid epoxides as a prophylactic or therapeutic agent (Falck et al., 2011; Adebesin et al., 2019). Such mimics and sEH inhibitors are in clinical development by several companies but none are available on the market (Lazaar et al., 2016; Hammock et al., 2021). However, there are a number of sEH inhibitors from natural sources that are commercially available such as Maca (also known as Peruvian ginseng, *Lepidium meyenii*) (Kitamura et al., 2017; Singh et al., 2020).

One of the sex-dependent observations in our study was related to lymphoid aggregate formation in the lung. Male sex in the *Fat-1* transgenic mouse had lesser number of aggregates as compared to their corresponding WT controls, which was further reduced in the presence of TPPU (Figures 3B,D). While we observed a similar trend for the female mice these differences did not reach significance. Lymphoid aggregates, which can be composed of T cells, B cells and dendritic cells (van der Strate et al., 2006) play different roles in different disease models such as COPD and *tuberculosis*. For example, in a chronic cigarette smoke-induced murine COPD model, lymphoid aggregates are associated with adverse outcomes and thus pathological, whereas in a *tuberculosis* model induced by the *Mycobacterium tuberculosis*, it is found to be a host defense mechanism (Hertz et al., 2020; Tam et al., 2020). To our knowledge sex-differences in lymphoid aggregate formation in the lung has not been fully explored. A recent study found that females are more susceptible to lymphoid aggregate formation as compared to males, and this was further confirmed by ovariectomy in a smoke-induced COPD model (Tam et al., 2020). This is consistent with the previous reports indicating that women are more susceptible to develop COPD than men (Martinez et al., 2007; de Torres et al., 2009; Perez et al., 2020). Overall, our results are consistent with the previously published literature on the effects of agricultural dust in males; and the improved histopathological effects seen in the male sex are novel.

We observed an elevated alveolar cellularity (approximately two-fold), in wild-type female mice as compared to male mice. A study investigated the differences in alveolar macrophage proteome between males and females, and found several proteins associated with inflammation and interact with estrogen receptor to be expressed higher in females than males (Phelps et al., 2012). Another study looking at sex-related differences in lung inflammation showed a higher baseline for lung histology score and number of infiltrating cells into the lungs in females compared to males in sham-operated controls (Vegeto et al., 2010). Both studies are consistent with the histological finding we observed in female wild-type mouse lungs. Because males responded TPPU treatment better, this might be related to differences in fatty acid metabolism between males and females given fatty acid epoxides regulate alveolar cell influx, as shown in a study demonstrating that pharmacological modulation of fatty acid epoxides affect inflammatory cell influx to the lungs (Yang et al., 2015). In addition, SPMs also modulate macrophage chemotaxis, trans-endothelial migration and cytokine release from macrophages as reviewed by Haworth and Levy (2007). More studies are needed to dissect differences in fatty acid metabolism between males and females.

It is well accepted that sex-specific differences that result in different biological responses between males and females stem from sex hormones. These differences affect storage and distribution of lipids thereby affect free fatty acid availability between sexes. Stable isotope studies report that ARA and DHA contribute more to blood lipids in women than in men and that there are differences in the conversion rate of fatty acids, for example conversion of ALA to omega-3 fatty acids is higher in women than in men (Decsi and Kennedy, 2011). Also, preclinical studies show that reproductive hormones affect the enzymes involved in the biosynthesis of fatty acids. Another study found that sex-related differences in COPD might be related in part to the increased production of leukotoxin-diol (linoleic acid derived diol) by goblet cell P450 and sEH activities (Balgoma et al., 2016). Overall, our results suggest a mechanism that 1) availability of free fatty acids from the elevated tissue levels of ω-3 acids in *Fat-1* mice are sex-dependent and 2) females have different conversion rates of fatty acids as compared to males and this might lead to changes in gene expression of chemokines and cytokines, thereby affecting alveolar macrophage recruitment into the lung and thus creating sex-dependent host defense mechanism within the *Fat-1* genotype.

In addition to dietary strategies aimed at promoting the healthful benefits of ω-3 PUFA, therapeutic strategies leveraging the endogenous repair SPM pathways to promote inflammation resolution and repair hold great clinical promise. For example, many investigations have identified positive outcomes in ameliorating lung inflammation/disease *via* therapeutic administration of SPM (Croasdell et al., 19502016; Seki et al., 19502010; Aoki et al., 2008; Haworth et al., 2011; Hsiao et al., 2013; Miyata and Arita, 2015; Kim et al., 2016), including our own previous studies identifying beneficial effects of the DHA-derived SPM maresin-1 in reducing the lung inflammatory effects of acute and repetitive organic dust exposure (Nordgren et al., 2013; Nordgren et al., 2015). While SPM can potently inhibit inflammation while promoting tissue repair, these bioactive metabolites are quickly deactivated by subsequent metabolism (Lopez-Vicario et al., 2015; Basil and Levy, 2016). Thus, another therapeutic strategy to leverage these endogenous inflammation resolution pathways is to combine ω-3 PUFA supplementation with pharmacologic inhibition of enzymes responsible for the deactivation of SPM. One such strategy has been the use of inhibitors of the sEH enzyme to prevent the deactivation of the cytochrome P450 family of SPM, thereby potentiating their protective effects (Guedes et al., 2013; Ulu et al., 2013; Ulu et al., 2014; Yang et al., 2015; Ulu et al., 2016; Zhou et al., 2016; Goswami et al., 2017; Zhou et al., 2017; Yang et al., 2020; Zhang et al., 2020). Previous studies have found enhanced protective effects of ω-3 PUFA when used in combination with inhibitors of sEH such as TPPU (Wang et al., 2012; Lopez-Vicario et al., 2015; Yang et al., 2015; Zhou et al., 2016; Zhou et al., 2017). This enzyme deactivates the epoxide SPM (e.g., 19, 20-EDP; produced through CYP450 metabolism) into less active diol forms (e.g., 19 (20)-DiHDPA). A recent study in a model of metabolic disease identified that a sEH inhibitor enhanced the protective effects identified in *Fat-1* mice vs. WT mice (Lopez-Vicario et al., 2015), while previous studies also identify beneficial effects of sEH inhibition in murine models of acute lung injury (Zhou et al., 2017), pulmonary fibrosis (Zhou et al., 2016), asthma (Yang et al., 2015), and COPD (Wang et al., 2012). Corroborating these previous reports, when we utilized TPPU in the experiments described herein, we found that addition of TPPU lowered all outcomes examined in the DE-exposed animals, suggesting that TPPU not only enhanced the effects of ω-3 fatty acids as in *Fat-1* + DE + TPPU animals, but also showed efficacy independent of the *Fat-1* genotype. In addition, we observed a better response in the *Fat-1* male sex receiving the TPPU treatment and three-weeks DE exposure. Consistent with our results sex-specific differences have been reported in sEH null mice and sEH activity in other mouse disease models (Vanella et al., 2015; Wagner et al., 2017; Jamieson et al., 2020).

While our results support a beneficial effect of maintaining a low omega6:omega3 ratio in response to agricultural dust, omega-6 PUFAs and their metabolites also modulate inflammation and participate in inflammation resolution and tissue homeostasis. The SPMs derived from arachidonic acid, such as LXA4 and metabolites generated by the P450 pathway (i.e., EETs or epoxyeicosatrienoic acids) have been repeatedly shown to have anti-inflammatory effects (Levy and Serhan, 2003; Morisseau and Hammock, 2013). Most surprisingly, the ARA metabolites generated by the COX-2 pathway, such as PGE2 have protective effects in the lung despite their infamous proinflammatory notion, as shown in asthma and allergic airway inflammation (Vancheri et al., 2004; Herrerias et al., 2009). Airway epithelial cells are the major source of PGE2 production in the lung, and PGE2 protects against airway hyperresponsiveness to allergens by inhibiting leukotriene and thromboxane synthesis that cause bronchoconstriction and by reducing eosinophil recruitment, both of which are anti-inflammatory effects of PGE2. Similarly, in asthma, it has been shown that the homeostatic balance between the COX and LOX pathways metabolizing ARA are altered due to damaged epithelium in asthmatic airways. Given this, it has been proposed that the dysregulation of these pathways leads to an imbalance between PGE2 and PGD2/LT which is in part responsible for increased bronchoconstriction. Other roles attributed to PGE2 in the lung include inflammatory cell recruitment, eosinophil degranulation, bronchodilation, T-cell recruitment and differentiation and adhesion molecule expression as reviewed before (Vancheri et al., 2004).

With regards to sex-related differences in response to environmental stimuli, a study examined changes in gene expression in the lung associated with inflammation and immunity after ozone exposure (Cabello et al., 2015). This study found increased lung histological scores and increased infiltrating PMN in the female sex as compared to males after ozone exposure. Among control mice exposed to filtered air alone, females displayed about 5% difference in gene expression of genes related to chemokines and cytokines as compared to males. These genes included *Cxcl2* and *Ccl19* (involved in activation of macrophages), *Myd88* (involved in TLR activation), and *C4b* (associated with IL-6 response), all of which were associated with immune cell adhesion and recruitment. Considering the sex- and genotype-dependent differences in our model, we examined changes in gene expression as well. Since the most significant changes we observed in our model was in the male sex (as in *Fat-1* mice with TPPU), we focused our gene expression studies to male sex. NanoString gene expression analysis identified differentially regulated pathways consistent with our previous results (Dominguez et al., 2020; Ulu et al., 2021b) indicating that immune cell activation, cell proliferation, wound healing and transport pathways were altered following 3-weeks DE exposure. In an In-depth analysis both using STRING database protein-protein interaction and NanoString advanced analyses, we also identified changes in NFκBIA, response to macrophage colony stimulating factor, immune clearance, and neutrophil aggregation between the two genotypes (Figure 4). In addition, TPPU treatment affected distinct cellular processes such as T-cell differentiation in WT mice and regulation of neutrophil activation in the *Fat-1* genotype. This observed effect of TPPU is consistent with a previous report showing modulation of the Th1/Th17 response while elevating regulatory T-cells by TPPU in an arthritis model (Trindade-da-Silva et al., 2020). Evaluation of both upregulated and downregulated genes among the experimental groups were in the direction of inflammation resolution. For example, in the *Fat-1* genotype the observed differences in the downregulated genes were associated with cytokine production in macrophages, regulation of tight junctions and ECM disassembly, suggesting a move towards resolution of inflammation in tissue (Table 1). Cytokine production and tight junctions contribute significantly to the maintenance of epithelial cell integrity and mucosal immunity of the lung in response to environmental insults as well as pathogens (Kyd et al., 2001; Brune et al., 2015). Similarly, changes in ECM are important for recruitment of immune cells into and within the lung (Wight et al., 2017). Altogether, changes in these processes promoted by elevated levels of ω-3 fatty acids as in *Fat-1* transgenic mice might affect how the lung responds to DE exposure. Particularly with TPPU, we identified downregulation of genes involved in IL-33 mediated signaling, as well as IL-5, IL-13, and IL-6 secretion. Overall, these data identified differences in immune clearance between the two genotypes and TPPU appeared to contribute to T-cell differentiation and regulation of PMN activation in part by downregulating the IL-33-mediated signaling pathway. IL-33 has previously been reported as an important regulator of Th-2 immune response in allergic inflammation (Chan et al., 2019).

The studies described herein do have numerous limitations. As with all transgenic animal models, the use of the *Fat-1* mice to increase total body tissue levels of ω-3 PUFA and achieve an ideal ∼1:1 ratio of ω-6:ω-3 PUFA does not fully recapitulate the human condition. For example, differences in PUFA intakes are seen not only between individuals, but temporal fluctuations in diet tendencies for each individual also undoubtedly alter daily to monthly PUFA levels, and the impacts of this will vary across different tissues based on PUFA uptake kinetics (Katan et al., 1997; Raphael and Sordillo, 2013; Gurzell et al., 2014). As PUFA substrate utilized during inflammatory events likely comes from both tissue sources as well as circulating blood and associated inflammatory cell infiltration, the impacts of recent dietary intake versus long-term dietary patterns on PUFA substrate availability are difficult to ascertain. Also, there are numerous recognized health benefits of diets high in ω-3 PUFA, due at least partly to their role in the endogenous production of SPM that regulate inflammation resolution and repair activities (Titos et al., 19502011; Schwab et al., 2007; Serhan et al., 2009; Levy, 2010; Serhan, 2014). SPM are produced temporally during an inflammatory response; their levels increase within hours to days following an inflammatory insult, and their production is critical to inflammation catabasis, including promoting neutrophil clearance, reducing inflammatory cytokine production, activating M2-like pro-resolution macrophages, promoting regulatory T cell recruitment, and activating tissue repair (Widgerow, 2012; Basil and Levy, 2016). Deficiencies in SPM generation pathways have been identified in asthma, and SPM are decreased in lavage, sputum, and/or exhaled breath condensates of COPD and asthma patients compared to individuals without lung disease (Levy et al., 2007; Bhavsar et al., 2010; Croasdell et al., 2015). Therefore, an in-depth analysis of both ω -3 and ω -6 PUFA derived SPMs is necessary, and lipid metabolomics analyses of our data are currently underway in our laboratory.

Another limitation is that we have used a model of organic dust exposure that utilizes a sterile-filtered aqueous extract of environmental dusts that is intranasally instilled to mice in a saline solution. This model will not fully recapitulate the inhalant injury that is experienced by an individual working in a swine confinement facility, as it does not consider certain components, including live pathogens or gaseous components that have recognized respiratory impacts in these workers (Von Essen and Donham, 1999; Kirkhorn and Garry, 2000; Charavaryamath et al., 2005; Charavaryamath et al., 2008; Langley, 2011; May et al., 2012; Madsen et al., 2015; Nordgren and Bailey, 2016; Schneberger et al., 2017; Nordgren and Charavaryamath, 2018; Schneberger et al., 2021). It has been reported that airway inflammation in swine confinement workers differ from naïve subjects even after repetitive exposures, one displaying neutrophilic airway inflammation and the other neutrophilic and eosinophilic inflammation, respectively (Larsson et al., 1994; Von Essen and Romberger, 2003). We have characterized a number of components that are likely to be involved in neutrophilic inflammation. Proteases are one of the components of agricultural dust that has been shown to be in part responsible for this type of inflammation (Von Essen and Romberger, 2003; Romberger et al., 2015). Our results are consistent with neutrophilic inflammation observed in people.

In addition, since we delivered agricultural dust extract under light isoflurane anesthesia, the previously reported sex-related differences of isoflurane might confound some of the sex-related effects we observed in our study; however, given all the mice in each group underwent this light anesthesia the significant differences between the groups would still stand.

Compelling data indicate the importance of having a balanced ω-6:ω-3 PUFA ratio for optimal health (∼1:1 is considered ideal), yet the typical Western diet has a ratio of ∼10–20:1 (Simopoulos, 2008; Patterson et al., 2012). This imbalance is considered a driving or compounding factor in chronic inflammatory diseases, with many pro-inflammatory lipids formed from the ω-6 PUFA arachidonic acid, such as leukotrienes, thromboxanes and prostaglandins (Calder, 2006; Calder, 2010; Patterson et al., 2012; Calder, 2013; Barden et al., 2016). It was recently reported that Veterans with COPD and an occupational history that include agricultural work had an ω-6:ω-3 PUFA ratio of ∼50:1 (Hanson et al., 2017), underscoring the potential vulnerability of this population. Supplementation with ω-3 PUFA has demonstrated health benefits in clinical studies of cystic fibrosis, COPD, and other lung diseases (Strasser et al., 1985; Pontes-Arruda et al., 2008; Giudetti and Cagnazzo, 2012; Oliver and Watson, 2013). Thus, increasing ω-3 FA intake, possibly in combination with a reduced intake of ω-6 PUFA, could be an accessible and effective means of preventing and ameliorating airway disease in patients with inflammatory lung diseases such as those caused by agricultural dust exposures. Taken together, our investigations utilizing the *Fat-1* mouse model in conjunction with sEH inhibition highlight the potentials of targeting repair/resolution pathways therapeutically to promote lung protection from environmental dust exposures, and also highlight differences based on sex in the protectiveness offered by these interventions. In the case of agriculture workers who are chronically inhaling inflammatory dusts, these outcomes hold promise for improving lung health outcomes *via* both limiting lung inflammation but also promoting repair following injury in this vulnerable population.

## Data Availability

The original contributions presented in the study are included in the article/Supplementary Material, further inquiries can be directed to the corresponding author.
