# Supplementary figures and images for: Sex-Specific Differences in Resolution of Airway Inflammation in Fat-1 Transgenic Mice Following Repetitive Agricultural Dust Exposure
Source: Front Pharmacol. 2022 Jan 13;12:785193. doi: 10.3389/fphar.2021.785193 (PMC8793679; doi:10.3389/fphar.2021.785193)

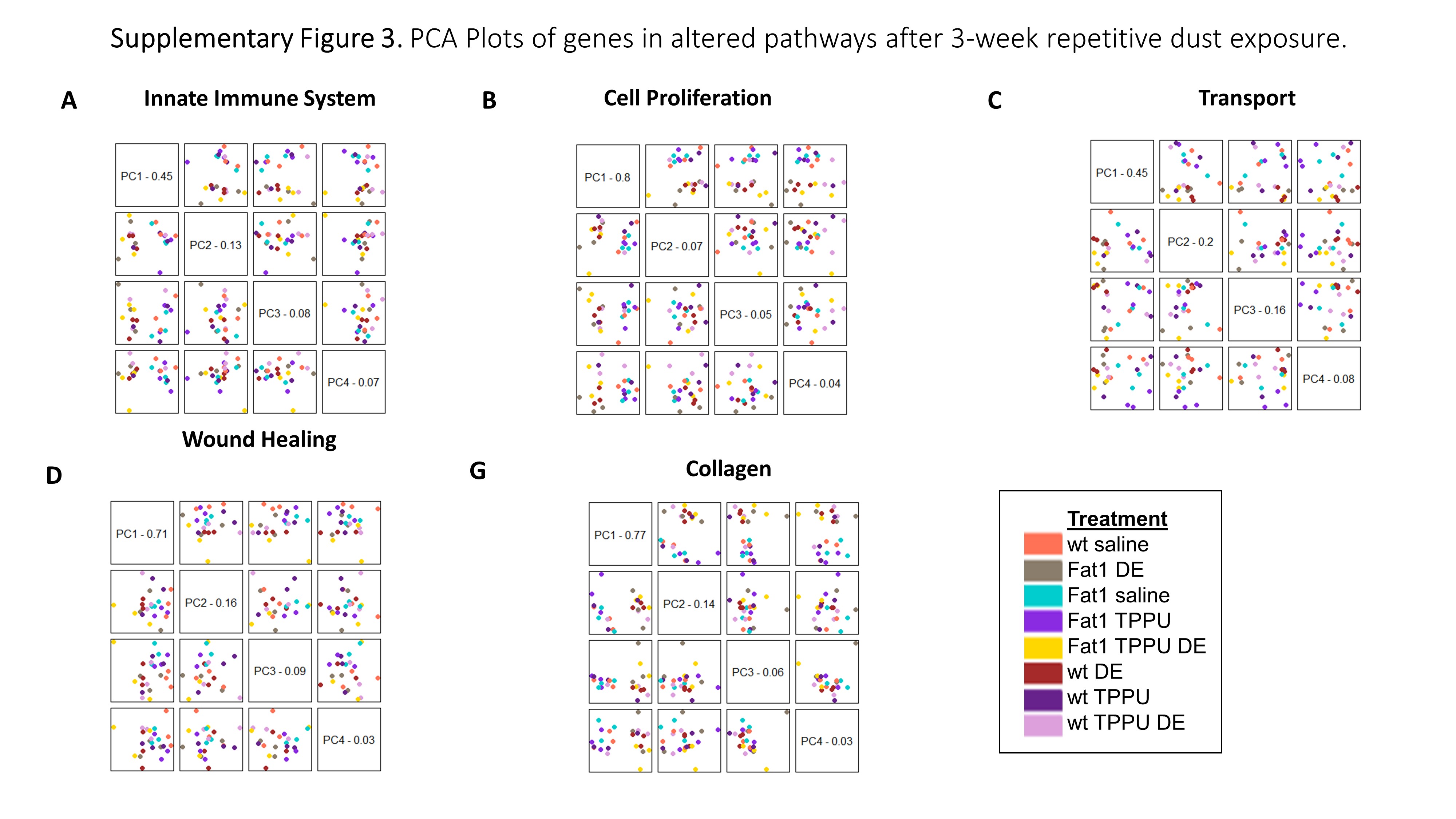

Supplement: Supplementary file 1 [file Image3.JPEG]

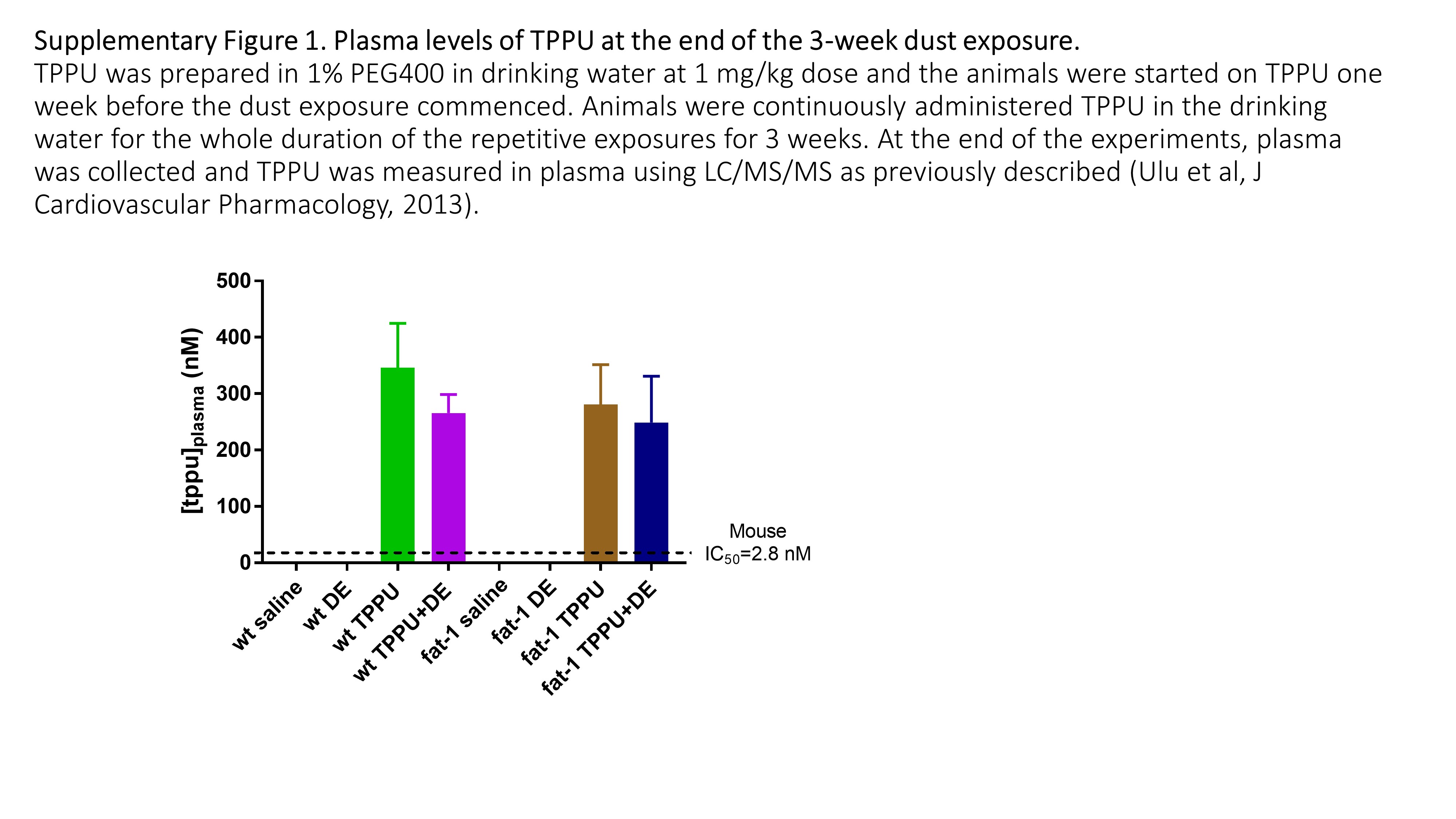

Supplement: Supplementary file 3 [file Image1.JPEG]

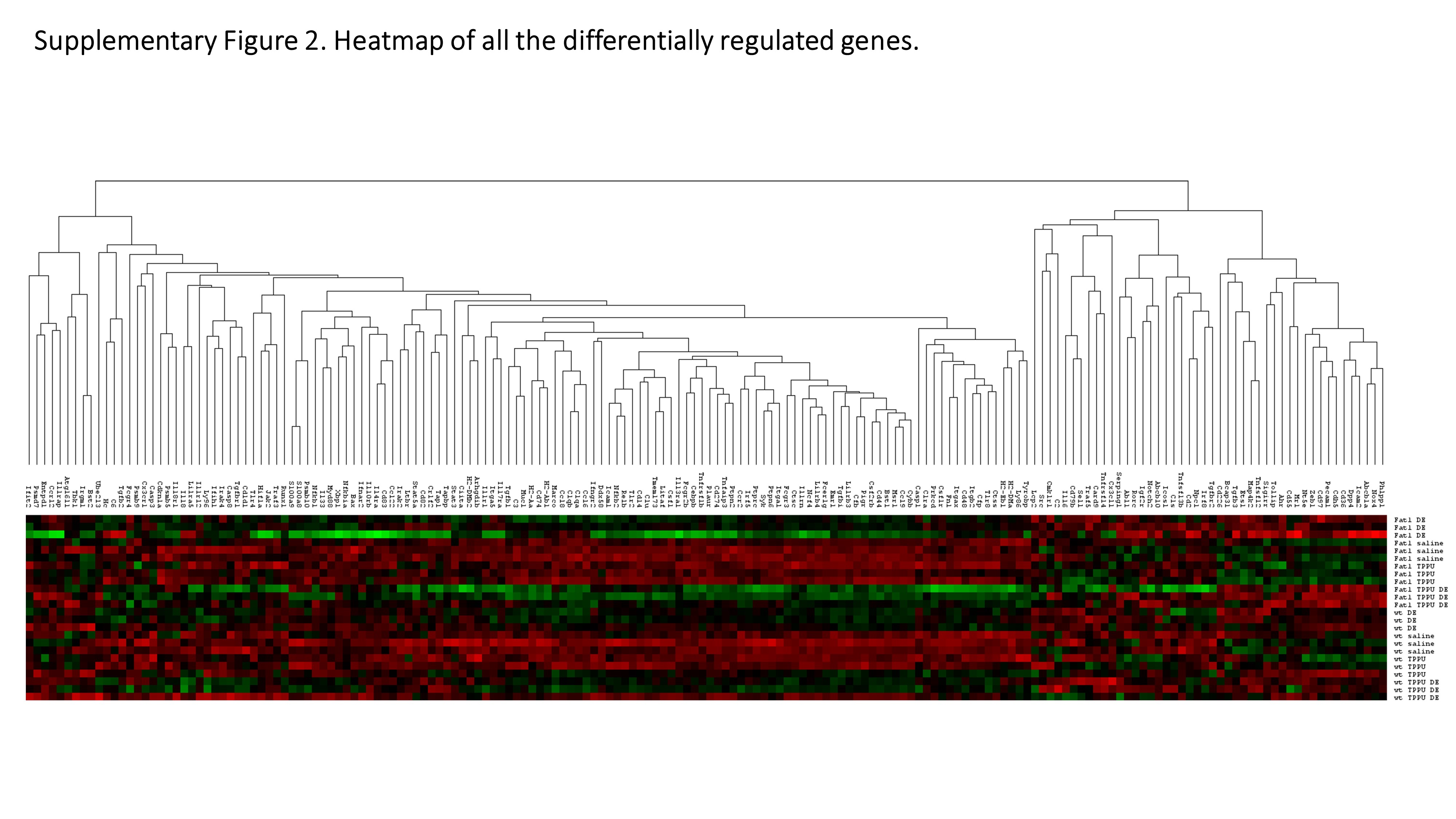

Supplement: Supplementary file 4 [file Image2.JPEG]
