# Supplementary material for: Sex-Specific Differences in Resolution of Airway Inflammation in Fat-1 Transgenic Mice Following Repetitive Agricultural Dust Exposure
Source: Front Pharmacol. 2022 Jan 13;12:785193. doi: 10.3389/fphar.2021.785193 (PMC8793679; doi:10.3389/fphar.2021.785193)
Supplement: Supplementary file 2 [file Table1.DOCX]

**Table 1.** STRING Database analysis of the differentially regulated genes and their association with biological pathways.

|  | **Upregulated genes** | **STRING Database biological process** | **P** | **Downregulated genes** |  | **STRING Database biological**  **process** |  | **P** |
| --- | --- | --- | --- | --- | --- | --- | --- | --- |
| WT DE vs. WT saline | 74 | Pos reg hematopoietic stem cell migration  Neg reg hippocampal neuron apoptotic process  Immune cell clearance | <1.0e-16 | 2 |  | n/a |  | n/a |
| Fat-1 DE vs. WT saline | 99 | Pos reg cellular response to macrophage colony stimulating factor  Pos reg hematopoietic stem cell migration  Pos reg growth factor dependent skeletal muscle proliferation  Neutrophil aggregation  Antigen processing and presentation of exogenous peptide via MHC Class I | <1.0e-16 | 13 |  | Protein localization to bicellular tight junction  Regulation of macrophage cytokine production  Regulation of ECM disassembly |  | 2.11e-06 |
| Fat-1 DE vs. Fat-1 saline | 80 | Pos reg cellular response to macrophage colony stimulating factor  Pos reg hematopoietic stem cell migration  Neutrophil aggregation | <1.0e-16 | 24 |  | B-1 B cell homeostasis  IL-17 production  Protein localization to bicellular tight junction |  | 7.86e-08 |
| Fat-1 DE vs. WT DE | 25 | Immune complex clearance  Neutrophil aggregation | <1.0e-16 | 8 |  | Neutrophil aggregation  Immune complex clearance |  | n.s. |
| WT DE vs. WT DE + TPPU | 1 (*LCP2*) | Positive regulation of T-cell differentiation | n/a | 0 |  | n/a |  | n/a |
| Fat-1 DE+TPPU vs. Fat-1 DE | 31 | Positive regulation of PMN activation  Synapse pruning | <1.0e-16 | 9 |  | IL-33 mediated signaling pathway  Pos regulation of IgG, IL-5, IL-13 and IL-6 secretion |  | 0.000471 |
| Fat-1 DE+TPPU vs. WT DE+TPPU | 43 | Pos reg hematopoietic stem cell migration  Neg reg hippocampal neuron apoptotic process  Antigen processing and presentation of exogenous peptide via MHC Class I  Macrophage colony-stimulating factor signaling pathway | <1.0e-16 | 4 |  | Cell autonomous role of endothelial GTP cyclohydrolase1 and tetrahydrobiopterin in blood pressure regulation |  | 0.109 |
