# Supplementary material for: Sex-Specific Differences in Resolution of Airway Inflammation in Fat-1 Transgenic Mice Following Repetitive Agricultural Dust Exposure
Source: Front Pharmacol. 2022 Jan 13;12:785193. doi: 10.3389/fphar.2021.785193 (PMC8793679; doi:10.3389/fphar.2021.785193)
Supplement: Supplementary file 5 [file DataSheet1.PDF]

SUPPLEMENTARY TABLE 1

Group Comparison	Upregulated genes			Downregulated genes		
	Gene	Fold change	p value	Gene	Fold change	p-value
**WT saline *versus* WT + DE**	Tgfbi	1.18	2.96E-05	Tnfsf12-mRNA	-0.358	0.0153
	Emr1	1.07	5.10E-05	Ets1-mRNA	-0.265	0.0542
	Bst1	0.985	7.96E-05			
	Itgax	1.52	0.000104			
	Lilrb3	1.3	0.000143			
	Ctss	0.885	0.000271			
	Ccr2	0.802	0.000284			
	Cybb	1.29	0.00034			
	Itgb2	1.03	0.000367			
	Fn1	1.4	0.000393			
	Cfb	2.26	0.00041			
	Cd74	0.628	0.000416			
	Cd44	0.966	0.000536			
	Muc1	0.596	0.000548			
	C1qb	0.858	0.000658			
	C1qa	0.912	0.000674			
	H2-Dma	0.56	0.000718			
	Pigr	1.8	0.000787			
	Ccl6	1.18	0.00108			
	Csf1r	0.538	0.00113			
	Tlr8	0.862	0.0012			
	Ccl9	2.28	0.00189			
	Fcgr3	0.932	0.00197			
	Ptprc	0.681	0.00216			
	Csf2rb	1.25	0.00233			
	H2-Ab1	0.601	0.00237			
	Ptpn6	0.471	0.00304			
	Ctsc	0.718	0.00312			
	Tgfb1	0.501	0.00313			

SUPPLEMENTARY TABLE 1

Group Comparison	Upregulated genes			Downregulated genes		
	Gene	Fold change	p value	Gene	Fold change	p-value
	Itga5	0.579	0.00326			
	Fcer1g	0.791	0.00331			
	Lilrb4	1.28	0.00366			
	Cd48	0.574	0.0039			
	Irf5	0.772	0.00489			
	Ccl8	1.78	0.00573			
	Fcgr2b	0.884	0.00636			
	C1ra	0.428	0.00682			
	Marco	1.6	0.00689			
	Itgal	0.696	0.00689			
	Cx3cr1	0.513	0.00704			
	Ncf4	0.79	0.00724			
	Syk	0.836	0.00854			
	Cd274	0.826	0.00943			
	H2-Aa	0.379	0.00991			
	Npc1	0.369	0.0108			
	Lilra5	0.767	0.0117			
	Tgfbr1	0.528	0.0131			
	Cfp	0.589	0.0134			
	Cd2	0.471	0.0138			
	H2-Eb1	0.426	0.0161			
	Il1rn	1.58	0.0175			
	Tyrobp	0.508	0.0176			
	Arhgdib	0.373	0.0189			
	C3	0.729	0.019			
	Clu	0.586	0.0196			
	Il17ra	0.423	0.0197			
	Tnfsf13b	0.411	0.022			
	Ddx58	0.219	0.0231			

SUPPLEMENTARY TABLE 1

Group Comparison	Upregulated genes			Downregulated genes		
	Gene	Fold change	p value	Gene	Fold change	p-value
	Icosl	0.502	0.0285			
	Ly86	0.416	0.031			
	Tnfrsf1b	0.753	0.034			
	Tgfbr2	0.256	0.0341			
	Tgfb2	0.336	0.0354			
	Il18	0.452	0.0363			
	Ciita	0.428	0.0374			
	Cd14	1.19	0.0387			
	Il1r1	0.412	0.0408			
	Casp8	0.266	0.0412			
	Il13ra1	0.315	0.0421			
	Plaur	0.496	0.0461			
	Irf8	0.423	0.0462			
	Prkcd	0.293	0.047			
	Casp3	0.305	0.0472			
	C6	0.331	0.0473			
**WT saline *versus* FAT-1 + DE**	Ccr2	0.997	2.95E-05	Map4k2	-0.927	0.00106
	Fcgr2b	1.57	4.07E-05	Tnfsf12	-0.491	0.00192
	Bst1	1.03	4.95E-05	Ets1	-0.422	0.00446
	Il1rn	3.21	6.19E-05	Tgfb3	-0.417	0.0125
	Lilrb3	1.4	6.69E-05	Ahr	-0.335	0.0234
	Tnfaip3	1.45	8.11E-05	Cd36	-0.438	0.035
	Cd274	1.44	9.10E-05	Abcb1a	-0.457	0.038
	Emr1	1	0.000107	Lcp2	-0.279	0.0433
	Cfb	2.59	0.000107	Dpp4	-0.36	0.0452
	Ncf4	1.29	0.000113	Stat5b	-0.366	0.0456
	Cd44	1.12	0.000135	Zeb1	-0.284	0.0483

SUPPLEMENTARY TABLE 1

Group Comparison	Upregulated genes			Downregulated genes		
	Gene	Fold change	p value	Gene	Fold change	p-value
	Csf2rb	1.67	0.000172	Pecam1	-0.269	0.0494
	Ctsc	1	0.000176	Cdh5	-0.297	0.0515
	Lilrb4	1.81	0.000189	Icam2	-0.48	0.0116
	Tgfbi	0.982	0.000196			
	Fcgr3	1.2	0.000208			
	Il13ra1	0.675	0.000221			
	Cd74	0.668	0.000231			
	Clu	1.05	0.000258			
	Fcer1g	1.06	0.000274			
	Pigr	1.95	0.000382			
	Cybb	1.28	0.000385			
	Cebpb	0.575	0.00042			
	Ccl9	2.72	0.000421			
	Csf1	0.864	0.000474			
	Plaur	0.993	0.000512			
	Ddx58	0.376	0.000521			
	Irf5	1.01	0.000553			
	Ciita	0.788	0.000623			
	Ptpn2	0.709	0.000624			
	Tmem173	1.18	0.000658			
	Itgax	1.25	0.000667			
	Nfkbia	0.609	0.00076			
	Tnfrsf1b	1.32	0.000894			
	Ptprc	0.758	0.000906			
	Litaf	0.808	0.000978			
	H2-Ab1	0.67	0.000986			
	Cd14	2.12	0.00102			
	Ptpn6	0.537	0.00107			
	Il4ra	0.816	0.0012			

SUPPLEMENTARY TABLE 1

Group Comparison	Upregulated genes			Downregulated genes		
	Gene	Fold change	p value	Gene	Fold change	p-value
	Il33	0.896	0.00123			
	Syk	1.09	0.00125			
	H2-Aa	0.493	0.00155			
	Tgfb1	0.546	0.0016			
	Irak2	0.522	0.00178			
	Muc1	0.516	0.00185			
	Ctss	0.702	0.00202			
	Cd83	1.14	0.0022			
	Cfp	0.767	0.00223			
	Il17ra	0.583	0.00242			
	Cd48	0.608	0.00254			
	S100a8	1.59	0.00265			
	Il10rb	0.308	0.00269			
	Ccl22	1.56	0.0027			
	Tyrobp	0.677	0.0028			
	C1ra	0.484	0.00286			
	Fn1	1.05	0.00419			
	Il1r1	0.612	0.00434			
	Hif1a	0.479	0.00469			
	H2-Dma	0.439	0.00482			
	Bax	0.431	0.005			
	C3	0.905	0.00516			
	Tgfbr1	0.611	0.00526			
	Tlr2	1.15	0.00586			
	Ccl8	1.77	0.00591			
	Xbp1	0.416	0.00594			
	S100a9	1.59	0.00611			
	Ccl6	0.933	0.00614			
	Tlr8	0.691	0.0064			

SUPPLEMENTARY TABLE 1

Group Comparison	Upregulated genes			Downregulated genes		
	Gene	Fold change	p value	Gene	Fold change	p-value
	Tap1	0.435	0.00682			
	Itgb2	0.702	0.00753			
	Csf1r	0.403	0.00924			
	H2-DMb2	0.65	0.00942			
	Nfkb2	0.9	0.00964			
	Arhgdib	0.419	0.00982			
	C1qb	0.592	0.0103			
	Itgal	0.643	0.0113			
	H2-Eb1	0.453	0.0113			
	Prkcd	0.38	0.013			
	Ifngr2	0.338	0.015			
	Casp3	0.385	0.0155			
	Myd88	0.526	0.0158			
	Jak2	0.491	0.0171			
	C1qa	0.577	0.0174			
	Itga5	0.433	0.0208			
	Tlr4	0.382	0.0223			
	Casp8	0.293	0.0268			
	Icam1	0.564	0.0277			
	Stat3	0.252	0.0301			
	Runx1	0.388	0.0313			
	Relb	0.777	0.0314			
	Crlf2	0.348	0.0344			
	Ltbr	0.207	0.0384			
	Nfkb1	0.295	0.0415			
	Ifit2	0.366	0.0421			
	Stat5a	0.358	0.0476			
	Cd82	0.213	0.0505			
	Tapbp	0.252	0.0532			

SUPPLEMENTARY TABLE 1

Group Comparison	Upregulated genes			Downregulated genes		
	Gene	Fold change	p value	Gene	Fold change	p-value
**WT + DE *versus* Fat-1 + DE**	Nfkbia	0.703	0.000209	Ahr	-0.406	0.00784
	Cd83	1.2	0.00147	Cd2	-0.415	0.0268
	Tnfaip3	0.983	0.00255	Cx3cr1	-0.397	0.028
	Il33	0.814	0.00262	Map4k2	-0.538	0.0352
	Xbp1	0.443	0.00386	Npc1	-0.29	0.0379
	Il4ra	0.673	0.00508	Irf8	-0.427	0.0446
	S100a9	1.53	0.00787	Tgfb3	-0.321	0.0466
	S100a8	1.35	0.00837	Abcb1a	-0.43	0.0493
	Cebpb	0.362	0.0131			
	Csf1	0.54	0.0144			
	Il1rn	1.63	0.0148			
	Ptpn2	0.452	0.0152			
	Il10rb	0.236	0.0154			
	Jak2	0.496	0.0161			
	Il13ra1	0.36	0.0221			
	Tmem173	0.693	0.0238			
	Fcgr2b	0.689	0.0259			
	Ccrl2	0.717	0.0261			
	Bax	0.317	0.0295			
	Cd274	0.617	0.0393			
	Plaur	0.496	0.0449			
	Sell	0.539	0.046			
	Litaf	0.418	0.0525			
	Irak2	0.288	0.0534			
	Clu	0.468	0.0547			

SUPPLEMENTARY TABLE 1

Group Comparison	Upregulated genes			Downregulated genes		
	Gene	Fold change	p value	Gene	Fold change	p-value
**Fat-1 + DE *versus* Fat-1 saline**	Tnfsf12	0.587	0.000409	Tnfaip3	-1.57	3.54E-05
	Map4k2	0.945	0.000902	Nfkbia	-0.823	4.23E-05
	Ets1	0.51	0.00104	Cfb	-2.76	5.50E-05
	Nt5e	0.54	0.00235	Il1rn	-3.05	0.000105
	Tgfb3	0.474	0.00548	Lilrb4	-1.92	0.000109
	Abcb1a	0.644	0.00566	Bst1	-0.936	0.000133
	Ahr	0.419	0.00635	Csf1	-0.979	0.000147
	Stat5b	0.518	0.00729	Fcgr2b	-1.38	0.000162
	Icam2	0.503	0.00866	Ccr2	-0.836	0.000181
	Mr1	0.435	0.00903	Pigr	-2.11	0.000185
	Cdh5	0.41	0.0103	Clu	-1.08	0.000204
	Phlpp1	0.62	0.0103	Cd44	-1.04	0.000274
	Cd97	0.478	0.0166	Ptpn2	-0.76	0.000338
	Abl1	0.55	0.0194	Cd14	-2.4	0.00035
	Cd226	0.49	0.0226	Ccl9	-2.77	0.000353
	Rorc	0.631	0.0319	Cebpb	-0.581	0.000379
	Dpp4	0.386	0.0331	Cd274	-1.24	0.000395
	Cmklr1	0.341	0.0353	Plaur	-1.01	0.000424
	Pecam1	0.284	0.0395	Fcgr3	-1.1	0.000483
	Zeb1	0.298	0.0396	Cd83	-1.33	0.000622
	Traf5	0.458	0.0415	Fcer1g	-0.962	0.000666
	Notch2	0.364	0.0483	Il33	-0.957	0.000706
	Fcgrt	0.404	0.0516	Ncf4	-1.05	0.000753
	Cd36	0.396	0.0534	Bax	-0.547	0.000791
				Ddx58	-0.359	0.000792
				Litaf	-0.818	0.000887
				Csf2rb	-1.39	0.000929
				Tmem173	-1.13	0.000981
				Emr1	-0.774	0.00109

SUPPLEMENTARY TABLE 1

Group Comparison	Upregulated genes			Downregulated genes		
	Gene	Fold change	p value	Gene	Fold change	p-value
				Ctsc	-0.81	0.00121
				Tlr2	-1.43	0.00122
				C3	-1.06	0.00166
				Irf5	-0.885	0.00168
				Tnfrsf1b	-1.21	0.00175
				C1qb	-0.764	0.00178
				Lilrb3	-0.965	0.00179
				Nfkb2	-1.13	0.00198
				Cybb	-1.04	0.0021
				Tgfbi	-0.744	0.00221
				Il4ra	-0.742	0.00254
				Il13ra1	-0.5	0.00283
				Cfp	-0.741	0.0029
				C1qa	-0.752	0.0033
				Ctss	-0.657	0.0033
				Ccl8	-1.91	0.0035
				Syk	-0.95	0.00356
				Cdkn1a	-0.993	0.00371
				Cd74	-0.48	0.00373
				H2-Ab1	-0.551	0.00445
				H2-Aa	-0.427	0.00456
				Ccl6	-0.972	0.00465
				S100a8	-1.46	0.00502
				Myd88	-0.626	0.00547
				Ifngr2	-0.395	0.00571
				Tyrobp	-0.606	0.00609
				C1ra	-0.431	0.0064
				S100a9	-1.48	0.00971
				Itgax	-0.867	0.00992

SUPPLEMENTARY TABLE 1

Group Comparison	Upregulated genes			Downregulated genes		
	Gene	Fold change	p value	Gene	Fold change	p-value
				Il10rb	-0.252	0.0106
				Ccl22	-1.27	0.0109
				Il1r1	-0.528	0.0111
				Il17ra	-0.461	0.0114
				Relb	-0.936	0.0119
				Nox4	0.752	0.0122
				Ciita	-0.513	0.0127
				Ptpn6	-0.375	0.013
				Xbp1	-0.365	0.0133
				Icam1	-0.634	0.015
				Hif1a	-0.394	0.0155
				Muc1	-0.359	0.0197
				Psmb5	-0.361	0.0201
				Tgfbr1	-0.475	0.0232
				Cd48	-0.422	0.024
				Tlr4	-0.353	0.0327
				Irak2	-0.322	0.0334
				Crlf2	-0.35	0.0337
				Il18r1	-0.464	0.0389
				Itgb2	-0.513	0.0398
				Ptprc	-0.396	0.0489
				Psmb10	-0.322	0.0512
**Fat-1 + DE *versus* Fat-1 + DE + TPPU**	Cx3cr1	0.661	0.000861	Xbp1	-0.381	0.0103
	Cd2	0.623	0.00204	Il33	-0.55	0.0286
	Prkcd	0.486	0.00227	Psmd7	-0.188	0.0308
	H2-Dma	0.453	0.00349	Nfkbia	-0.339	0.0336
	Itgb2	0.779	0.00352	Ccrl2	-0.676	0.0343

SUPPLEMENTARY TABLE 1

Group Comparison	Upregulated genes			Downregulated genes		
	Gene	Fold change	p value	Gene	Fold change	p-value
	Fn1	0.994	0.00582	Entpd1	-0.295	0.0445
	H2-Eb1	0.494	0.00647	Il1rn	-1.27	0.0489
	Itgax	0.91	0.00705	Psmb7	-0.239	0.053
	Csf1r	0.413	0.00713	Il1rap	-0.35	0.0553
	C1qb	0.614	0.0079			
	Itga4	0.784	0.00835			
	Lcp2	0.375	0.00939			
	Ly86	0.508	0.00986			
	Irf8	0.55	0.0123			
	Cd48	0.466	0.0129			
	Npc1	0.346	0.0156			
	Tlr8	0.561	0.0186			
	C1qa	0.542	0.0231			
	Cd79b	0.659	0.0251			
	Ctss	0.464	0.0269			
	Lilra5	0.651	0.0271			
	Cfp	0.499	0.0278			
	Fcgr4	0.483	0.0291			
	Traf5	0.483	0.0326			
	Il16	0.449	0.033			
	Ccl6	0.658	0.0404			
	Src	0.201	0.0423			
	Ptprc	0.401	0.0449			
	H2-DMb2	0.471	0.0454			
	H2-Aa	0.277	0.0481			
	Abl1	0.449	0.0504			
